# Targeting c-IAP1, c-IAP2, and Bcl-2 Eliminates Senescent Glioblastoma Cells Following Temozolomide Treatment

**DOI:** 10.3390/cancers13143585

**Published:** 2021-07-17

**Authors:** Christian Schwarzenbach, Larissa Tatsch, Juliana Brandstetter Vilar, Birgit Rasenberger, Lea Beltzig, Bernd Kaina, Maja T. Tomicic, Markus Christmann

**Affiliations:** Department of Toxicology, University Medical Center, Obere Zahlbacher Str. 67, D-55131 Mainz, Germany; schwarzenbach@uni-mainz.de (C.S.); latatsch@uni-mainz.de (L.T.); jbrandst@uni-mainz.de (J.B.V.); rasebi00@uni-mainz.de (B.R.); lea.beltzig@uni-mainz.de (L.B.); kaina@uni-mainz.de (B.K.)

**Keywords:** inhibitor of apoptosis (IAP), alkylating drugs, malignant glioma, senescence

## Abstract

**Simple Summary:**

Despite extensive research, malignant glioma remains the most aggressive and fatal type of brain tumor. Following resection, therapy is based on radiation concomitant with the methylating agent temozolomide (TMZ), followed by adjuvant high-dose TMZ. In previous work, we showed that following TMZ exposure, most glioma cells evade apoptosis and enter a senescent state and are thereby protected against anticancer therapy. Senescent cells may escape from senescence, contributing to the formation of recurrences or can induce the senescence-associated secretory phenotype (SASP), which may impact therapy success. Therefore, direct targeting of senescent cells might be favorable to improve the effect of TMZ-based anticancer therapy. Here we show that during TMZ-induced senescence in glioblastoma cells, the antiapoptotic factors c-IAP2 and Bcl-2 are responsible for the prevention of cell death and that inhibition of these factors by BV6 and venetoclax effectively kills senescent glioblastoma cells.

**Abstract:**

Therapy of malignant glioma depends on the induction of O^6^-methylguanine by the methylating agent temozolomide (TMZ). However, following TMZ exposure, most glioma cells evade apoptosis and become senescent and are thereby protected against further anticancer therapy. This protection is thought to be dependent on the senescent cell anti-apoptotic pathway (SCAP). Here we analyzed the factors involved in the SCAP upon exposure to TMZ in glioblastoma cell lines (LN-229, A172, U87MG) and examined whether inhibition of these factors could enhance TMZ-based toxicity by targeting senescent cells. We observed that following TMZ treatment, c-IAP2 and Bcl-2 were upregulated. Inhibition of these SCAP factors using non-toxic concentrations of the small molecule inhibitors, BV6 and venetoclax, significantly increased cell death, as measured 144 h after TMZ exposure. Most importantly, BV6 and venetoclax treatment of senescent cells strongly increased cell death after an additional 120 h. Moreover, Combenefit analyses revealed a significant synergy combining BV6 and venetoclax. In contrast to BV6 and venetoclax, AT406, embelin, and TMZ itself, teniposide and the PARP inhibitor pamiparib did not increase cell death in senescent cells. Based on these data, we suggest that BV6 and venetoclax act as senolytic agents in glioblastoma cells upon TMZ exposure.

## 1. Introduction

Among the different subtypes of brain cancer, glioblastomas (WHO grade IV) account for ~60% of high-grade gliomas [1]. Despite intensive efforts, patients have a dismal prognosis of 14.6 months median survival and a 2-year survival rate of less than 26.5% [2]. The current treatment of glioblastomas consists of maximum safe resection followed by radiotherapy with concomitant and adjuvant temozolomide (TMZ) [3]. TMZ exerts its cytotoxic effect by the induction of *O^6^*MeG, and subsequently by the formation of DNA double-strand breaks (DSBs), finally leading to apoptosis [4,5,6]. However, in addition to apoptosis, TMZ strongly induces senescence in glioma cells [7,8].

Senescence was initially described as a permanent cell cycle arrest that limits the life span of cultured human fibroblasts [9]. Contrary to quiescence, which is defined as a temporary cell cycle arrest, senescence is not reversible in response to proliferative conditions (for review see [10,11,12,13]). In senescent cells, tumor-promoting and tumor-suppressing factors can be activated at the same time [14]. As a tumor-suppressing mechanism, the senescence-associated secretory phenotype (SASP), which is characterized by secretion of multiple immune factors, including interleukins, chemokines, growth factors, and matrix metalloproteinases [15,16,17], can reinforce the growth arrest by increasing ROS production and by enhancing the DNA damage response [17,18]. In addition, SASP induces an inflammatory response and activates immune cells, which eliminate senescent tumor cells [19,20]. On the other hand, SASP factors also act as potent tumor promoters, enhancing malignant tumorigenesis. Thus, the SASP can enhance the proliferation of neoplastic epithelial cells [21] and promote EMT [22,23] as well as tumor growth in vivo [24,25].

Several studies have indicated that cells can escape from genotoxin-induced senescence when the initial DNA damage stimuli are removed [26,27,28,29]. However, this matter is far from being resolved. Current knowledge suggests that senescence is highly dynamic and that an escape does not mediate a full conversion of the senescent phenotype back to the pre-senescent status [30]. In addition to well-established factors involved in cell cycle arrest, such as p53, p16, and Rb, or downregulation of mTOR, epigenetic alterations are also highly important for senescence. Thus, it was shown that the demethylases JMJD2C and LSD1 mediate escape from oncogene-induced senescence [31] and that the H3K9 histone methyltransferase Suv39h1 is important for maintaining therapy-induced senescence [32]. Moreover, single-cell experiments showed that tumor cells can restart proliferation and in parallel still show ß-Gal positivity [33]. Most importantly, it has been shown that oncogene-induced senescence can be overcome in melanocytes, turning them into tumor-initiating cells [34]. If this also holds true for TMZ exposure in glioma cells, then these cells, which accumulated genomic alterations during senescence, could contribute to the formation of recurrences. Killing of these senescent cells would be desirable for two reasons: firstly, to prevent potential formation of recurrences from senescent cells, and secondly, to reduce negative effects on non-tumorigenic cells induced by SASP [35]. Agents that kill senescent cells are called “senolytics”. Their discovery was based on the observation that senescent cells are resistant to apoptosis, due to upregulation of specific senescent cell anti-apoptotic pathways (SCAPs), which might be caused by senescence-associated mitochondrial dysfunction (SAMD) (for review see [36,37]).

Here, we analyzed whether the anti-apoptotic factors Bcl-2, Bcl-xL, c-IAP1, c-IAP2, XIAP, and Survivin play an essential role in the SCAPs induced by TMZ, and whether inhibition of these factors could enhance cytotoxicity by targeting senescent cells. Our data indicate that especially c-IAP2 and Bcl-2 play an important role in the TMZ-induced SCAPs. Thus, pharmacological inhibition of these factors strongly enhances cell death following TMZ and potentially improves the response of malignant glioma to TMZ-based therapy.

## 2. Materials and Methods

### 2.1. Cell Culture and Drug Treatment

The glioma cell lines LN-229 (RRID:CVCL_0393), A172 (RRID:CVCL_0131), and U87MG (RRID:CVCL_0022; since misidentified, it refers to as “glioblastoma of unknown origin”) were cultivated in Dulbecco’s minimal essential medium (DMEM) containing 10% fetal bovine serum (FBS) in a humidified atmosphere containing 5% CO_2_ at 37 °C. A172 and U87MG cells were purchased from Cell Line Service (Eppelheim, Germany), and the glioblastoma cell line LN-229 was obtained from LGC Standards (Wesel am Rhein, Germany). All three cell lines are proficient for p53 and deficient for MGMT. Immortalized human astrocytes were a gift from Timothy A. Chan (Memorial Sloan-Kettering Cancer Center, New York) and were previously described [38]. hTERT-immortalized RPE-1 cells were provided by ATCC. Cells were cultured in Dulbecco’s modified Eagle’s medium (DMEM) plus 10% FBS. Human colonic epithelial cells (HCEC) [39] were a gift from Prof. Jerry Shay (UT Southwestern Medical Center, Dallas) and cultured in RPMI with 5% FBS, supplemented with 25 ng/mL EGF (PeproTech), 1 µg/mL hydrocortisone, 10 µg/mL insulin, 2 µg/mL transferrin (Sigma), and 50 µg/mL G418 (Sigma). The cell lines were kept in culture for max. two months and were regularly checked for mycoplasm contamination using the VenorGEM classic detection kit (#11–1100) from Minerva Biologicals.

TMZ was a kind gift of Prof. Geoff Margison, Centre for Occupational and Environmental Health, University of Manchester, United Kingdom. It was solubilized in DMSO, diluted in distilled water immediately before use, and administeredat the indicated concentrations. BV6 (targets c-IAP1 and c-IAP2; CAS 1001600-56-1, Selleckchem), AT406 (targets c-IAP1 > XIAP; CAS 1071992-99-8, Selleckchem), venetoclax (targets Bcl-2; CAS 1257044-40-8, Selleckchem), and embelin (targets XIAP; CAS 550-24-3, Selleckchem) were solubilized in DMSO and used at the indicated concentrations.

### 2.2. Preparation of RNA, cDNA Synthesis, and Real Time PCR

Total RNA was isolated using the Nucleo Spin RNA Kit (Machery and Nagel, Düren, Germany). One µg total RNA was transcribed into cDNA (Verso cDNA Kit, Thermo Scientific), and qPCR was performed using the GoTaq^®^ qPCR Master Mix Protocol (Promega, Madison, USA) and the CFX96 Real-Time PCR Detection System (Biorad, München, Germany). In all experiments, PCR was performed in technical triplicates, and SEM showed intra-experimental variation. The analysis was performed using CFX Manager^TM^ Software. Non-transcribed controls were included in each run, expression was normalized to *GAPDH* and *ACTB*, and the untreated control was set to one. The specific primers are listed in Table A1.

### 2.3. Determination of Cell Death, Cell Cycle Progression, and Senescence

For monitoring TMZ-induced apoptosis, attached and detached cells were collected, and annexin V-FITC/propidium iodide (PI) double stained cells were analyzed by flow cytometry. To determine cell death and cell cycle distribution, attached and detached cells were collected and stained with PI and analyzed by flow cytometry using BD FACSCanto II. Senescence was measured by SA-β-Gal staining and flow cytometry-based C_12_FDG staining in attached cells as described [40]. Experiments were repeated at least three times; mean values ± SD are shown.

### 2.4. Preparation of Protein Extracts and Western Blot Analysis

Whole-cell and nuclear protein extracts were prepared as previously described [41]. Primary antibodies were diluted 1:500–1:1000 in 5% BSA and 0.1% Tween-TBS and incubated overnight at 4 °C. Peroxidase-coupled secondary antibodies were diluted 1:2000 and incubated 2 h at RT. The protein–antibody complexes were visualized by Pierce^®^ ECL Western Blotting Substrate (Thermo Fisher), and immunodetection was performed using the iBright CL1000 (Invitrogen) system. The specific antibodies are listed in Table A2.

### 2.5. Quantification and Statistical Analyses

The data were evaluated via Student’s *t*-test and were expressed as mean ± SD. * *p* ≤ 0.01 was considered statistically significant, ** *p* ≤ 0.01 very significant and *** *p* ≤ 0.001 highly significant. Statistics were performed using GraphPad Prism version 6.01 for Windows, GraphPad Software, La Jolla California USA. Combenefit software version 2.021 was downloaded at https://sourceforge.net/projects/combenefit/, accessed on 20 June 2021 and used to perform surface analyses of drug combinations to identify Loewe synergy [42].

## 3. Results

### 3.1. TMZ Induces Cell Death, Cell Cycle Arrest, and Senescence

**Figure 1:**
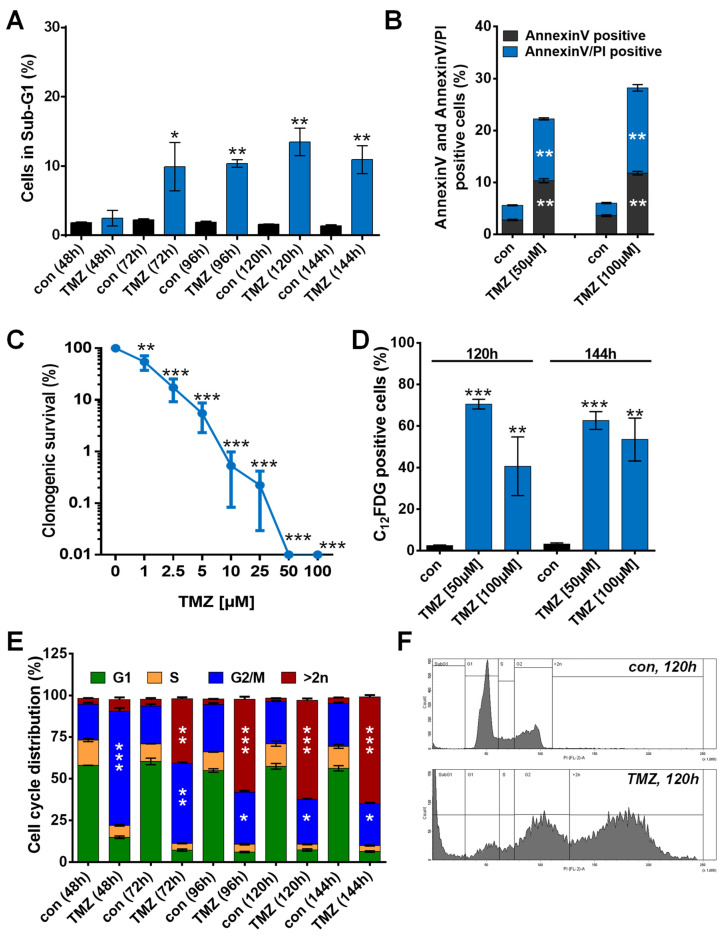
(**A**) Cell death was measured 48 to 144 h upon exposure to 50 µM TMZ by flow cytometry using PI staining in LN-229 cells. (**B**) Cell death was measured 120 and 144 h upon exposure to 50 µM TMZ by flow cytometry using Annexin V/PI staining in LN-229 cells. (**C**) Proliferation arrest was measured upon exposure up to 100 µM TMZ by colony formation assay (CFA). (**D**) Senescence was measured by flow cytometry using C_12_FDG staining 120 and 144 h after exposure to 50 or 100 µM TMZ. (**E**) Cell cycle distribution was measured 48 to 144 h upon exposure to 50 µM TMZ by flow cytometry using PI staining in LN-229 cells. (**F**) Representative histograms showing the appearance of cells harboring > 2n DNA 120 h after TMZ (50 µM) exposure. (**A**–**E**) Experiments were performed in triplicate, and differences between treatment and control were statistically analyzed using Student’s *t*-test (not labeled = not significant, * *p* < 0.1, ** *p* < 0.01, *** *p* < 0.001). Concerning cell cycle distribution, at all the time points, a significant (***) decrease of cells in G1 phase was observed.

To identify SCAPs responsible for the resistance of senescent glioblastoma cells to TMZ, we initially used the MGMT-deficient and p53-proficient cell line LN-229. Exposure of LN-229 cells to 50 µM TMZ showed a time-dependent induction of cell death, which however did not exceed 15% within the first 144 h after treatment (Figure 1A). Cell death was executed by apoptosis and necrosis, as shown by annexinV/PI-staining (Figure 1B). Despite the low toxicity, clonogenic survival dropped < 20% at concentrations ≥ 2.5 µM and was completely abolished at 50 µM TMZ (Figure 1C), and more than 70% of the cells exhibited a senescent phenotype as determined by C_12_FDG staining (Figure 1D). Importantly, higher TMZ concentrations (100 µM) did not enhance cell death or the frequency of senescent cells. Upon TMZ exposure, senescence was associated with an accumulation of cells in the G2-phase and a strong formation of polyploid cells, indicating inaccurate cell division (Figure 1E,F).

**Figure A1:**
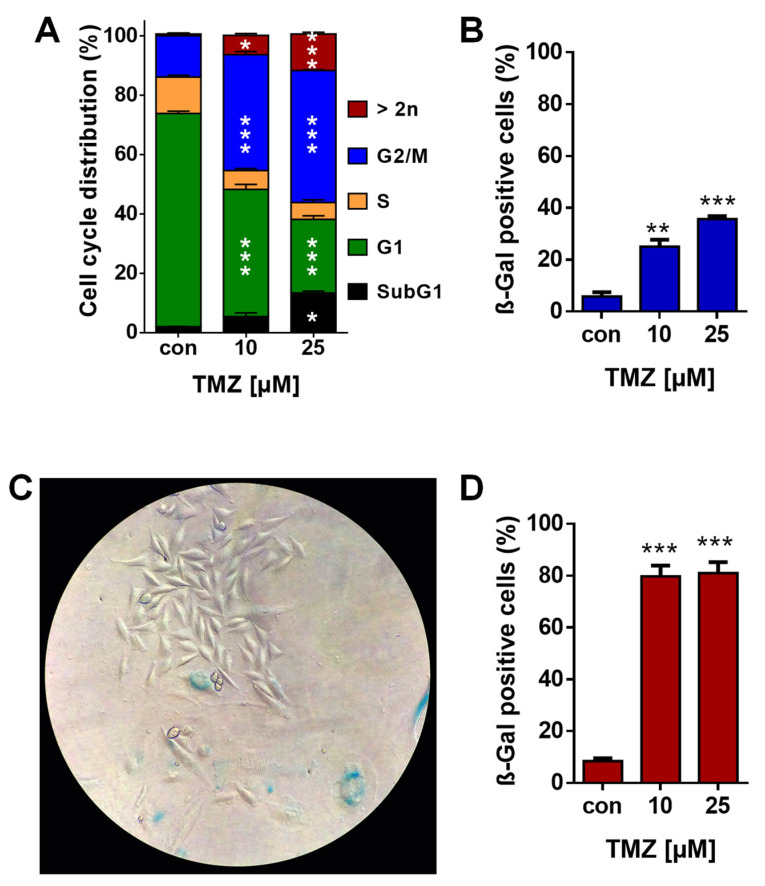
(**A**) Cell death and cell cycle distribution were measured 144 h upon exposure to 10 and 25 µM TMZ by flow cytometry using PI staining in LN-229 cells. (**B**) Senescence was measured by ß-Gal staining 144 h after exposure to 10 or 25 µM TMZ. (**C**) Microscopical image showing ß-Gal negative colony-forming cells and separated ß-Gal positive cells 144 h after exposure to 10 µM TMZ. (**D**) Experiments presented under B were re-evaluated, counting colonies as single cells. (**A**,**B**,**D**) Experiments were performed in triplicate, and differences between treatment and control were statistically analyzed using Student’s *t*-test (not labeled = not significant, * *p* < 0.1, ** *p* < 0.01, *** *p* < 0.001).

It is important to note that even low concentrations of TMZ (1–25 µM) induce a linear increase in the activation of the DNA damage response, cell death, and senescence [43,44]. However, the frequency of senescent cells was only 25% at 10 µM and 35% at 25 µM TMZ (Figure A1A,B). The low amount of β-Gal positive cells is presumably due to the fact that at low TMZ concentrations, a small number of cells was able to evade senescence, keeping their proliferating capacity, and form colonies at late times after TMZ treatment (144 h). Normally, measurement of senescent cells using β-Gal assay and C_12_FDG staining takes these colonies into account as separate cells. Thus, the clonogenic assay indicates how many cells are senescent at the time of analysis (e.g., 144 h), but not at which frequency the cells enter senescence after TMZ treatment, thereby underestimating the senescence level. To take this into account, we re-analyzed the data, considering individual colonies as a single cell derived from a senescence-evading cell (Figure A1C,D).

Using this approach, the estimated senescence level was significantly higher. This further indicates that, upon treatment with low TMZ concentrations, induction of senescence is the predominant trait in TMZ-exposed cells. It is conceivable that at late time points, which have to be chosen in order to assess SCAPs and identify potential senolytics, already a low frequency of senescence-evading cells would overgrow the senescent population. To omit bias caused by senescence-evading cells, a concentration of 50 µM TMZ was chosen for further experiments.

### 3.2. TMZ Induces Upregulation of Anti-Apoptotic Factors

**Figure 2:**
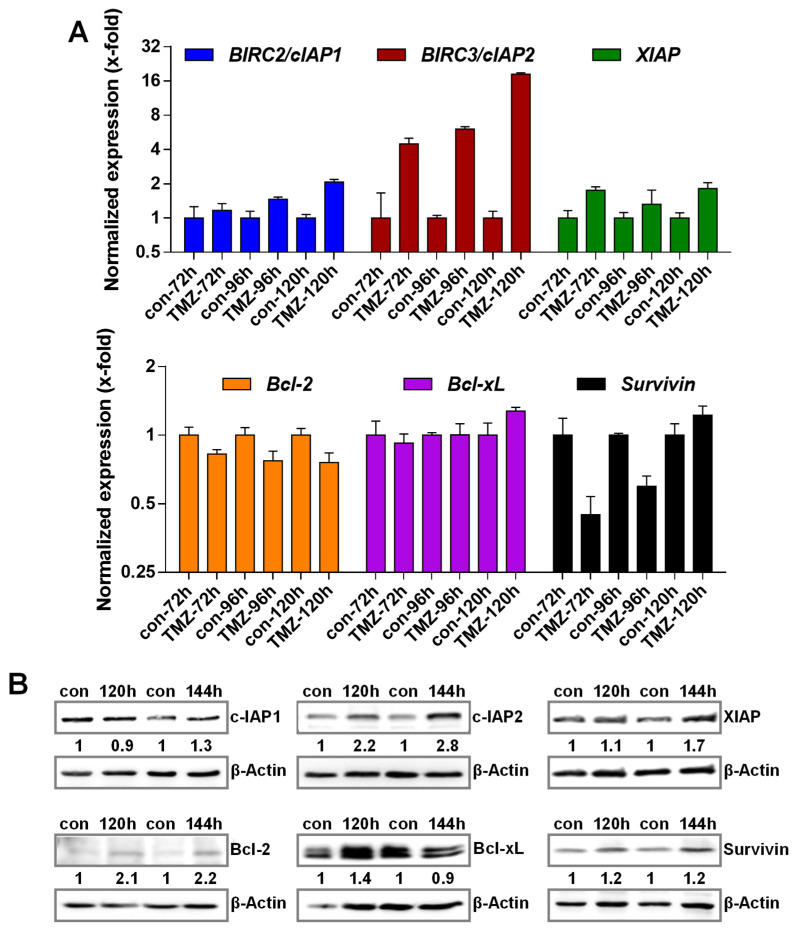
(**A**) LN-229 cells were treated with 50 µM TMZ; 72–120 h later, expression of *c-IAP1, c-IAP2, XIAP, Bcl-2, Bcl-xL*, and *BIRC5/Survivin* mRNA was measured by PCR. (**B**) LN-229 cells were treated with 50 µM TMZ; 120 and 144 h later, expression of Bcl-2, Bcl-xL, c-IAP1, c-IAP2, XIAP, and Survivin protein was measured by immunodetection.

Important players involved in the protection against apoptosis are factors that can block the activation of different executive caspases. Among them, especially members of the Bcl-2 family and the inhibitor of apoptosis (IAP) members have already been associated with SCAPs. To analyze whether one or multiple of these factors act as SCAPs in TMZ-induced senescence, we analyzed the expression of these factors in response to TMZ exposure. The data indicate a strong transcriptional activation of *c-IAP2* and a weak induction of *c-IAP1* and *XIAP* (Figure 2A).

For *Bcl-2* and *Bcl-xL*, no transcriptional alterations were observed, whereas *BIRC5*/*survivin* was repressed at early times after exposure. Similar to the data obtained at the transcriptional level, a strong increase in the expression of the c-IAP2 protein was observed (Figure 2B). Apart from c-IAP2, an induction of Bcl-2 was also observed at the protein level. For Bcl-xL, c-IAP1, Survivin, and XIAP, only minor alterations were visible.

### 3.3. Inhibition of Anti-Apoptotic Factors at Early Time Points Impacts TMZ-Induced Cell Death

**Figure A2:**
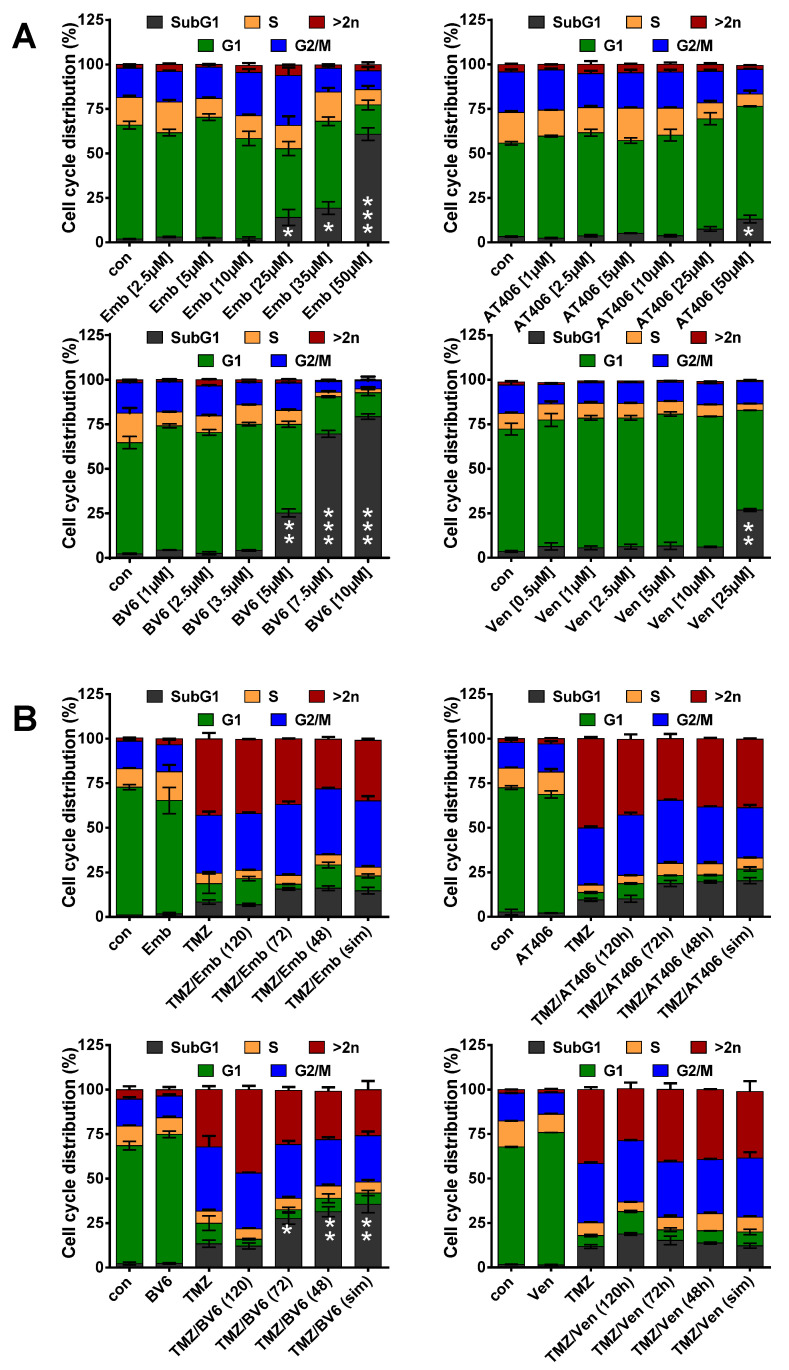
(**A**) LN-229 cells were treated with different concentrations of the XIAP inhibitor embelin, the c-IAP1 inhibitor AT406, the c-IAP1/c-IAP2 inhibitor BV6, and the Bcl 2 inhibitor venetoclax for 120 h. Cell death and cell cycle distribution were measured by flow cytometry using PI staining in LN-229 cells. (**B**) LN-229 cells were either non-treated (con) or treated with 50 µM TMZ for 120 h (TMZ). In addition, 5 µM embelin, 1 µM AT406, 1 µM BV6, or 5 µM venetoclax were added either simultaneously with TMZ (sim) or 48, 72, or 120 after TMZ exposure. Cell death and cell cycle distribution was measured by flow cytometry using PI staining in LN-229 cells. (**A**,**B**) Experiments were repeated at least three times; mean values ± SD are shown. Differences in the SubG1 fraction between control and TMZ treatment (**A**) and TMZ and inhibitor/TMZ treatment (**B**) were statistically analyzed using Student’s *t*-test (not labeled = not significant, * *p* < 0.1, ** *p* < 0.01, *** *p* < 0.001).

To test whether the factors of the IAP and Bcl-2 family might act as SCAPs, we analyzed whether inhibition of these proteins can cause death of senescent LN-229 cells. Therefore, we first identified a non-toxic concentration of the inhibitors embelin (targets XIAP), AT406 (targets c-IAP1 > XIAP), BV6 (targets c-IAP1 and c-IAP2), and venetoclax (targets Bcl-2) in LN-229 cells (Figure A2A). For embelin, toxicity started at 25 µM, for AT406 at 100 µM, for BV6 at 10 µM, and venetoclax was not toxic up to 50 µM. Next, we analyzed the impact of inhibition of these factors on cell death induction by TMZ. Therefore, cells were exposed to 50 µM TMZ for 144 h. The inhibitors were added either simultaneously (sim) or 48, 72, or 120 h after addition of TMZ to the medium (Figure A2B). Particularly, simultaneous treatment with TMZ and BV6 significantly increased toxicity. This effect gradually decreased by adding the inhibitor at later time points. In contrast, venetoclax, embelin, and AT406 treatment did not significantly affect TMZ-induced cell death. The data indicate that c-IAP2 is an important player in protection against TMZ-induced cell death and thereby supports the formation of the senescent phenotype.

### 3.4. Inhibition of Anti-Apoptotic Factors Kills Senescent LN-229 Cells

**Figure 3:**
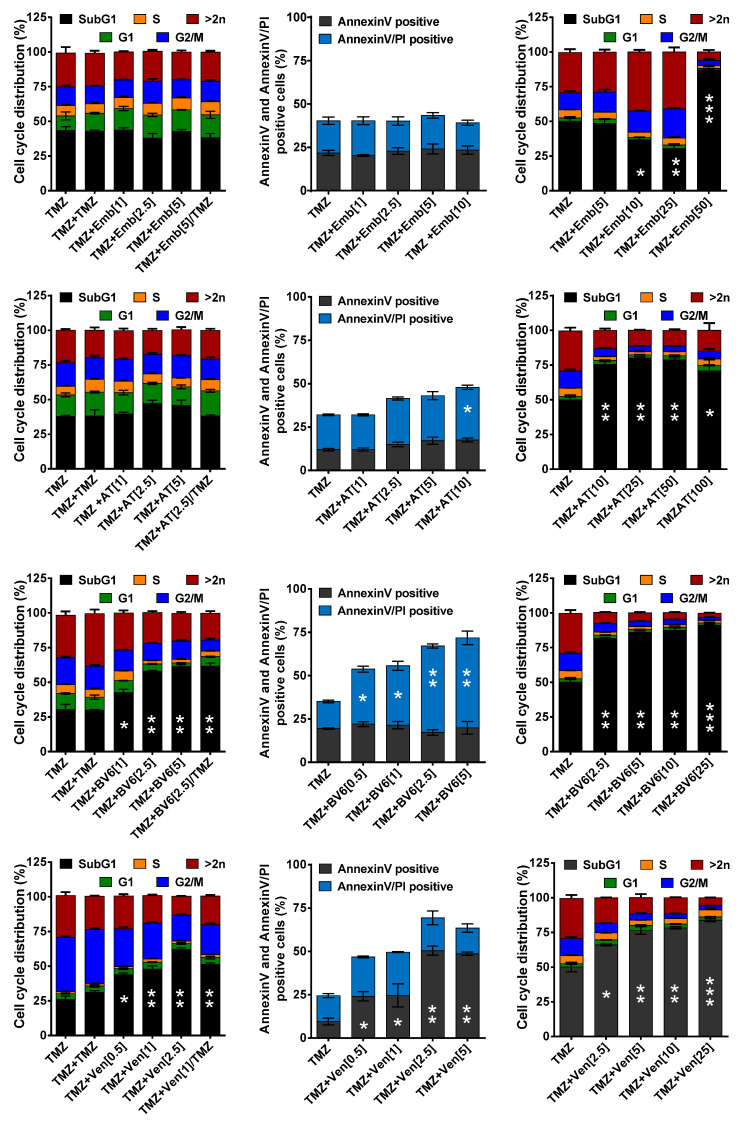
LN-229 cells were treated with 50 µM TMZ for 120 h. Thereafter the medium was changed, and the cells were cultivated for additional 120 h, either in the absence (TMZ), the presence of TMZ (TMZ + TMZ), the presence of different concentrations of the inhibitors only (TMZ+embelin, TMZ+AT406, TMZ+BV6, TMZ+venetoclax) or in the presence of the inhibitors combined with TMZ (TMZ+inhibitor/TMZ). **Left panel:** After 240 h in total, cell death and cell cycle distribution were measured by flow cytometry using PI staining in LN-229 cells. **Middle panel:** After total of 240 h, cell death was measured by flow cytometry using AnnexinV/PI staining in LN-229 cells. **Right panel:** After 240 h, cell death and cell cycle distribution were measured by flow cytometry using PI staining in LN-229 cells. In all cases, experiments were repeated at least three times; mean values ± SD are shown. Differences in the SubG1 fraction between inhibitor/TMZ treatment and TMZ alone were statistically analyzed using Student’s *t*-test (not labeled = not significant, * *p* < 0.1, ** *p* < 0.01, *** *p* < 0.001).

An intriguing question is whether the anti-apoptotic factors also play a crucial role in protection against cell death in senescent cells. Therefore, we analyzed whether inhibition of the potential SCAP factors can kill senescent cells. LN-229 cells were exposed to 50 µM TMZ and incubated for 120 h to induce senescence. Thereafter, the medium was replaced with fresh, FBS-containing medium, and the cells were either non-treated, again treated with TMZ, or treated with different concentrations of the inhibitors. In contrast to the second TMZ exposure, which had no impact on toxicity, treatment with low, non-toxic concentrations of the inhibitors BV6 and venetoclax significantly increased cell death (Figure 3, left column).

Thus, treatment with 5 µM BV6 increased the cell death frequency from 30 to 75% and treatment with venetoclax from 26 to > 60%. Opposite to BV6 and venetoclax, AT406 and embelin did not affect toxicity (Figure 3, left column). Similar results were observed analyzing cell death using AnnexinV/PI staining. Additionally, in this case, only BV6 and venetoclax dose-dependently enhanced cell death (Figure 3, middle column). Of note, BV6 predominantly increased necrosis/late apoptosis, whereas venetoclax exclusively increased the early-apoptotic cell fraction. To analyze whether higher concentrations of AT406 and embelin impact TMZ-induced toxicity, we repeated the experiments using embelin at a concentration of up to 50 µM. Interestingly, 10 and 25 µM embelin reduced TMZ-induced cell death, whereas 50 µM embelin enhanced the toxicity to >80% (Figure 3, right column); however, this embelin concentration was already highly toxic on its own (Figure A2A), indicating a non-specific effect. Similar to embelin, high concentrations of AT406 also clearly enhanced cell killing. Of note, the effect was similar in the concentration range 10–100 µM (Figure 3, right column), and AT406 showed moderate toxicity within this concentration range on its own (Figure A2A). Higher concentrations of venetoclax or BV6 dose-dependently enhanced cell death (Figure 3, right column); however, in the case of BV6, these high concentrations were toxic on their own, whereas for venetoclax, only the highest concentration (25 µM) was already toxic on its own (Figure A2A). Overall, the data indicate that non-toxic concentrations of BV6 and venetoclax and to a lesser extent of AT406 but not of embelin increased cell death in the TMZ-treated surviving (senescent) fraction and therefore could be used to eradicate senescent LN-229 cells upon TMZ treatment.

**Figure 4:**
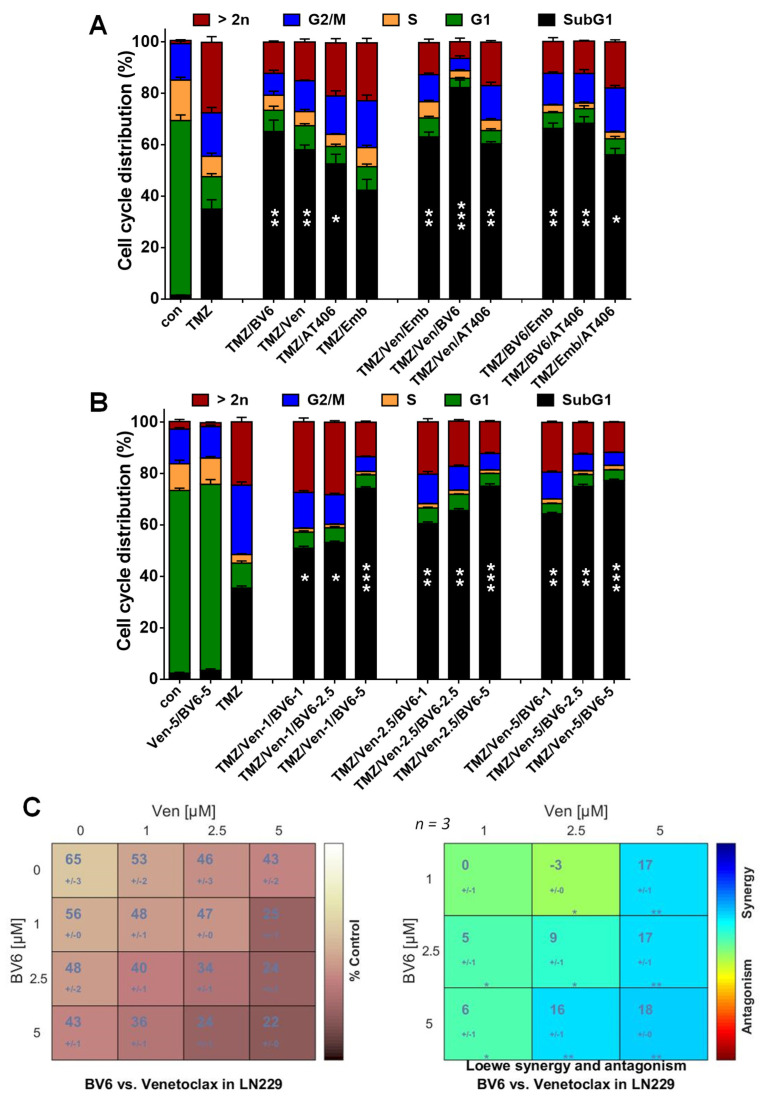
(**A**) LN-229 cells were treated with 50 µM TMZ for 120 h. Thereafter, the medium was changed, and the cells were either further cultivated for an additional 120 h in the absence (TMZ) or the presence of different concentrations of the inhibitors (5 µM embelin, 5 µM AT406, 2.5 µM BV6, 2.5 µM venetoclax) or in the presence of a combination of two inhibitors. As the control, LN-229 cells were cultivated for 120 h; thereafter, the medium was replaced, and the cells were further cultivated for 120 h (con). After a total of 240 h, cell death and cell cycle distribution were measured by flow cytometry using PI staining in LN-229 cells. (**B**) LN-229 cells were treated with 50 µM TMZ for 120 h. Thereafter the medium was changed, and the cells were either further cultivated for an additional 120 h in the absence (TMZ) or the presence of 1, 2.5, or 5 µM BV6 combined with 1, 2.5, or 5 µM venetoclax. As controls, cells were cultivated for 120 h; thereafter, the medium was replaced, and the cells were further cultivated for 120 h in the absence (con) or presence of the inhibitors (5 µM BV6/5 µM venetoclax). After a total of 240 h, cell death and cell cycle distribution were measured by flow cytometry using PI staining. (**A**,**B**) Experiments were repeated at least three times; mean values ± SD are shown. Differences in the SubG1 fraction between inhibitor/TMZ treatment and TMZ alone were statistically analyzed using Student’s *t*-test (not labeled = not significant, * *p* < 0.1, ** *p* < 0.01, *** *p* < 0.001). (**C**) Combenefit software was used to perform surface analyses to identify synergy between BV6 and venetoclax based on the data presented under (**B**).

Since the inhibitors target different anti-apoptotic factors, the question arose whether a combination of these inhibitors could further enhance cell death. Therefore LN-229 cells were exposed to 50 µM TMZ for 120 h to induce senescence. Thereafter, the medium was replaced with fresh, FBS-containing medium, and the cells were either non-treated or treated with the inhibitors alone or in combination. The data show that only the combination of BV6 and venetoclax was effective, enhancing the frequency of cell death from 35% (TMZ alone), 65% (BV6 + TMZ), and 60% (venetoclax + TMZ) to > 80% (BV6 + venetoclax + TMZ) (Figure 4A).

All other combinations showed only a minor effect. To substantiate this finding, we used additional BV6 and venetoclax concentrations to identify optimal conditions. Even the combination comprising the lowest concentrations (1 µM BV6/1 µM venetoclax) was sufficient to enhance the toxicity from 35% to 50% (Figure 4B). The strongest effect was achieved using the highest BV6 concentration (5 µM), culminating in cell death up to 80%. This was independent of the concentration of venetoclax used in the combination, showing the dominant effect of BV6. Only at low BV6 concentrations the toxicity was further enhanced by venetoclax (Figure 4B). Of note, even the highest combination (5 µM BV6/5 µM venetoclax) showed no significantly enhanced toxicity on its own. The statistical evaluation using Combenefit software confirmed this notion, showing a significant synergy between BV6 and venetoclax in triggering death of senescent LN-229 cells (Figure 4C).

**Figure A3:**
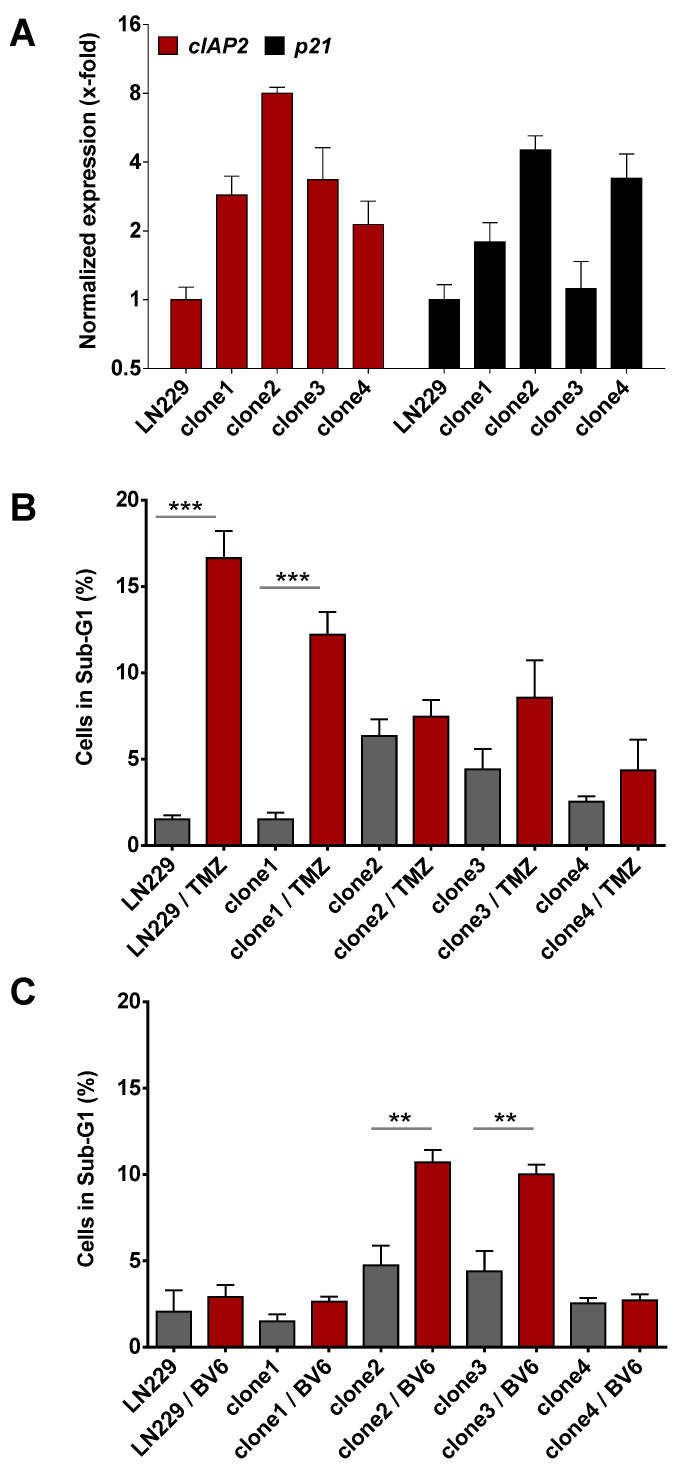
(**A**) LN-229 cells were treated with 50 µM TMZ. Four weeks later, four still proliferating colonies were picked, from which four cell clones were established. Expression of c-*IAP2* and p21 mRNA were measured by real-time qPCR. (**B**) LN-229 cells and the four cell clones were treated with 50 µM TMZ. Then, 144 h later, cell death was measured by flow cytometry using PI staining. (**C**) LN-229 cells and the four cell clones were treated with 2.5 µM BV6. Then, 144 h later, cell death was measured by flow cytometry using PI staining. (**B**,**C**) Experiments were repeated at least three times; mean values ± SD are shown. Differences in the SubG1 fraction between control and TMZ treatment (**B**) and between con and BV6 treatment (**C**) were statistically analyzed using Student’s *t*-test (not labeled = not significant, ** *p* < 0.01, *** *p* < 0.001).

As shown previously, senescent tumor cells repress important repair pathways [8]. Therefore, it is anticipated that these cells accumulate DNA damage during senescence and may represent an important trigger for the formation of recurrences upon escape from senescence [35]. To test whether senescence-escaping or senescence-evading cells can be targeted by BV6, we established four cell clones that still proliferate after TMZ exposure. Of note, the frequency at which these clones were built was ~ 0.02%. Interestingly, all of the clones maintained an increased expression of c-IAP2 and p21 (Figure A3A), while only one clone was still responsive to TMZ (Figure A3B). A significant enhancement of cell death was observed upon BV6 treatment in two of these clones (Figure A3C), indicating that some of the clones that survived the TMZ treatment still require c-IAP2 for survival.

### 3.5. Inhibition of Anti-Apoptotic Factors Kills Senescent A172 and U87MG Cells

**Figure 5:**
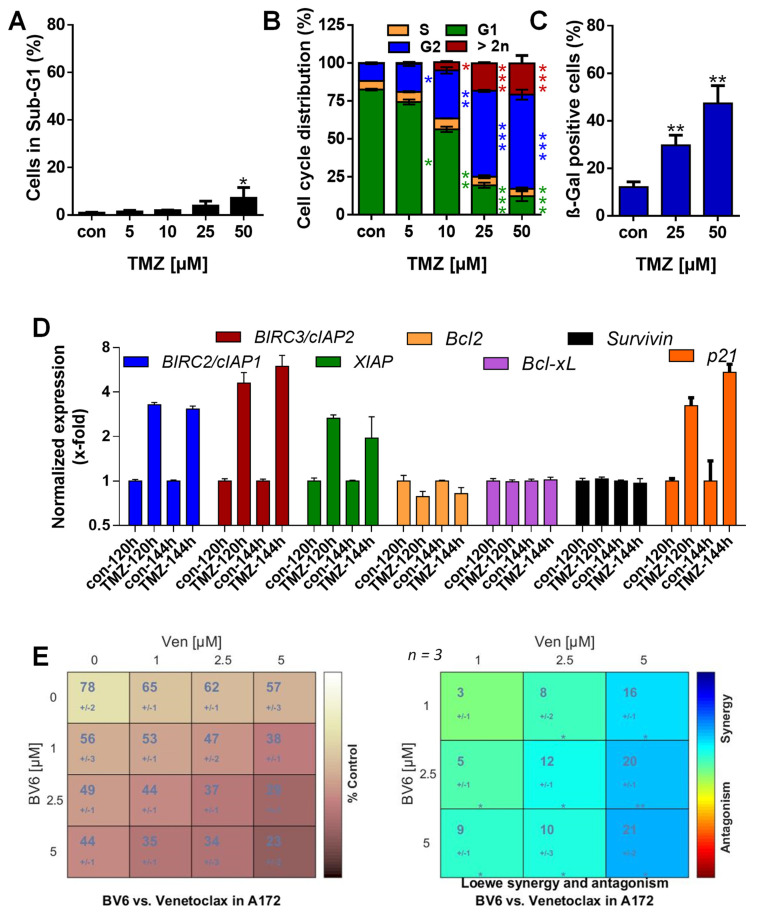
(**A**) Cell death was measured 120 h upon exposure to different concentrations of TMZ by flow cytometry using PI staining in A172 cells. (**B**) Cell cycle distribution was measured 120 h after exposure to different concentrations of TMZ by flow cytometry using PI staining in A172 cells. (**C**) Senescence was measured microscopically by detection of β-Gal positive cells 120 h after exposure to 25 and 50 µM TMZ. (**D**) A172 cells were treated with 50 µM TMZ. Then, 120 and 140 h later, expression of *c-IAP1, c-IAP2, XIAP, Bcl-2, Bcl-xL*, and *BIRC5/Survivin* mRNA was measured by PCR. (**E**) A172 cells were treated with 50 µM TMZ for 120 h. Thereafter, the medium was changed, and the cells were either further cultivated for an additional 120 h in the absence or presence of 1, 2.5, or 5 µM BV6 combined with 1, 2.5, or 5 µM venetoclax. After a total of 240 h, cell death distribution was measured by flow cytometry using PI staining in A172 cells, and Combenefit software was used to perform surface analyses to identify synergy. (**A**–**C**) Experiments were performed in triplicate, and differences between treatment and control were statistically analyzed using Student’s *t*-test (not labeled = not significant, * *p* < 0.1, ** *p* < 0.01, *** *p* < 0.001).

**Figure 6:**
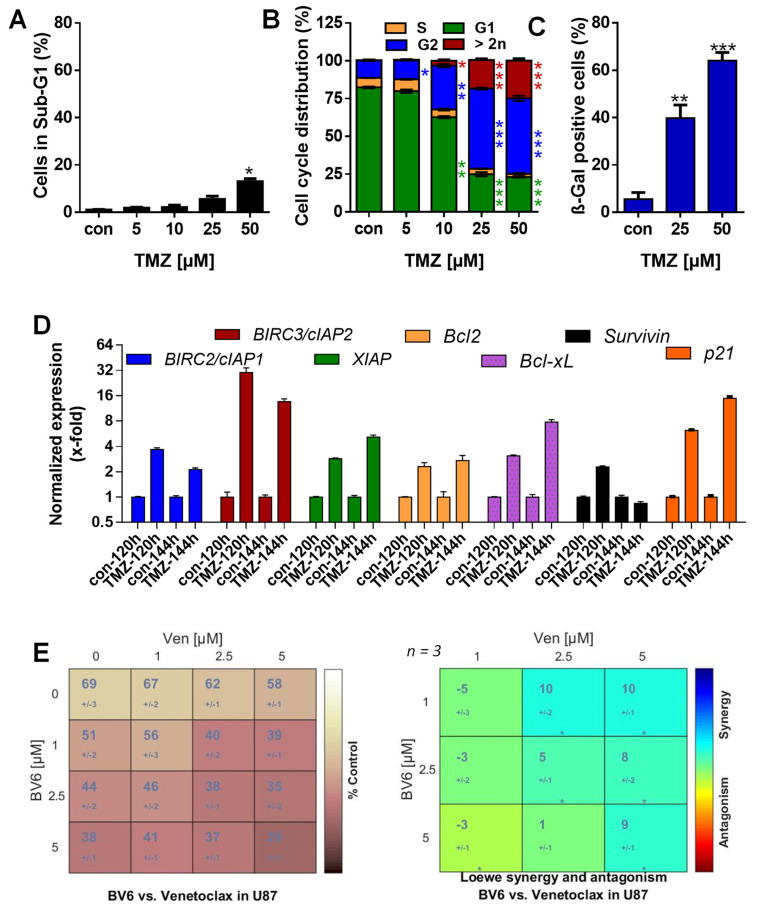
(**A**) Cell death was measured 120 h upon exposure to different concentrations of TMZ by flow cytometry using PI staining in U87MG cells. (**B**) Cell cycle distribution was measured 120 h upon exposure to different concentrations of TMZ by flow cytometry using PI staining in U87MG cells. (**C**) Senescence was measured microscopically by detection of β-Gal positive cells (left panel) or by flow cytometry using C_12_FDG staining (right panel) 120 h after exposure to 25 and 50 µM TMZ. (**D**) U87MG cells were treated with 50 µM TMZ. Then, 120 and 140 h later, expression of *c-IAP1, c-IAP2, XIAP, Bcl-2, Bcl-xL*, and *BIRC5/Survivin* mRNA was measured by PCR. (**E**) U87MG cells were treated with 50 µM TMZ for 120 h. Thereafter, the medium was changed, and the cells were either further cultivated for an additional 120 h in the absence or presence of 1, 2.5, or 5 µM BV6 combined with 1, 2.5, or 5 µM venetoclax. After a total of 240 h, cell death was measured by flow cytometry using PI staining in U87MG cells, and Combenefit software was used to perform surface analyses to identify synergy. (**A**–**C**) Experiments were performed in triplicate, and differences between treatment and control were statistically analyzed using Student’s *t*-test (not labeled = not significant, * *p* < 0.1, ** *p* < 0.01, *** *p* < 0.001).

Our data obtained in LN-229 cells suggests that particularly c-IAP2 and Bcl-2 act as SCAP factors in TMZ-induced senescence, and that inhibition of these factors could strongly kill these senescent cells. To test this hypothesis, we used two additional MGMT-deficient and p53-proficient glioblastoma cell lines (A172 and U87MG). In these cell lines, TMZ also induced predominantly senescence and not cell death. Thus, 120 h after exposure to 50 µM TMZ, cell death was < 10% in A172 (Figure 5A) and < 20% in U87MG cells (Figure 6A).

**Figure A4:**
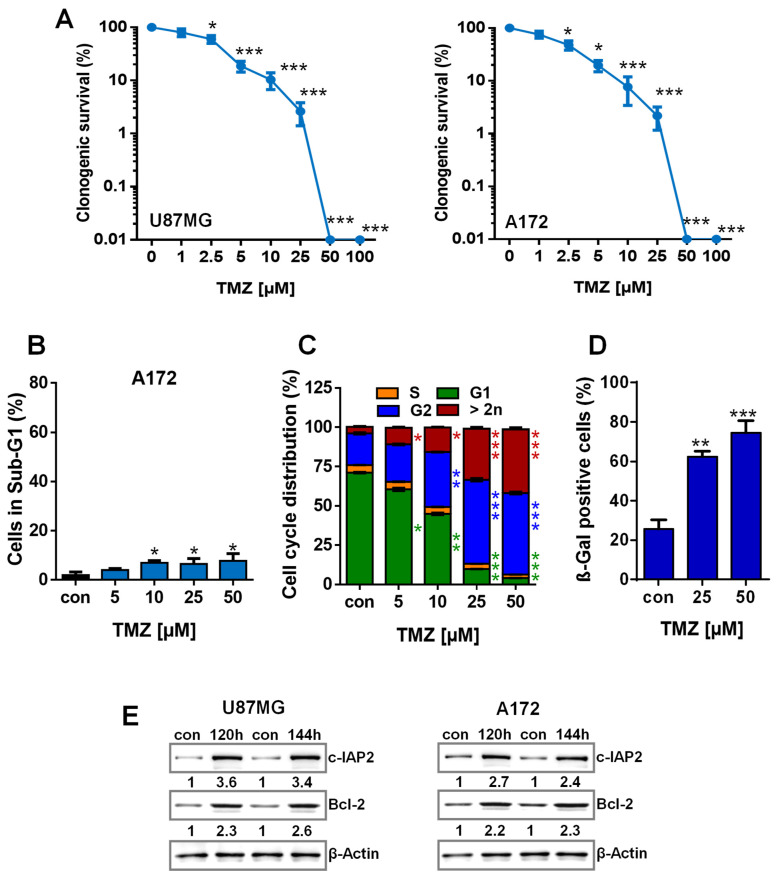
(**A**) Proliferation arrest and clonogenic survival was measured upon exposure up to 100 µM TMZ by colony formation assay in U87MG and A172 cells. Cell death (**B**) and cell cycle distribution (**C**) were measured 144 h upon exposure to 50 µM TMZ by flow cytometry using PI staining in A172 cells. (**D**) Senescence was measured by flow cytometry using SA-β-Gal staining 144 h after exposure to TMZ in A172 cells. (**E**) U87MG and A172 cells were treated with 50 µM TMZ. Then, 120 and 144 h later, expression of c-IAP2 and Bcl-2 protein was measured by immunodetection. (**A**–**D**) Experiments were performed in triplicate, and differences between treatment and control were statistically analyzed using Student’s *t*-test (not labeled = not significant, * *p* < 0.1, ** *p* < 0.01, *** *p* < 0.001).

Opposite to cell death, the arrest of the cells in the G2-phase and the appearance of cells with a DNA content >2n was significantly enhanced 120 h after TMZ exposure in both cell lines (Figure 5B and Figure 6B). The senescence frequency was ~ 45% in A172 and ~ 65% in U87MG cells (Figure 5C and Figure 6C). Moreover, clonogenic survival dropped to < 20% at concentrations ≥ 5 µM and was completely abolished at 50 µM TMZ (Figure A4A). We should note that A172 cells are slower-proliferating in comparison to LN-229 cells. Therefore, we also measured cell death and senescence 144 h after TMZ treatment; we observed no toxicity, but increased induction of senescence up to 75% (Figure A4B–D). Similar to LN-229 cells, transcriptional activation of *c-IAP1*, *c-IAP2*, and *XIAP* was observed in A172 and U87MG cells (Figure 5D and Figure 6D). Moreover, U87MG cells exhibited transcriptional activation of *Bcl-2* and *Bcl-xL* (Figure 6D). Importantly, also the protein levels of Bcl-2 and c-IAP2 were enhanced in both cell lines after TMZ exposure (Figure A4E).

**Figure A5:**
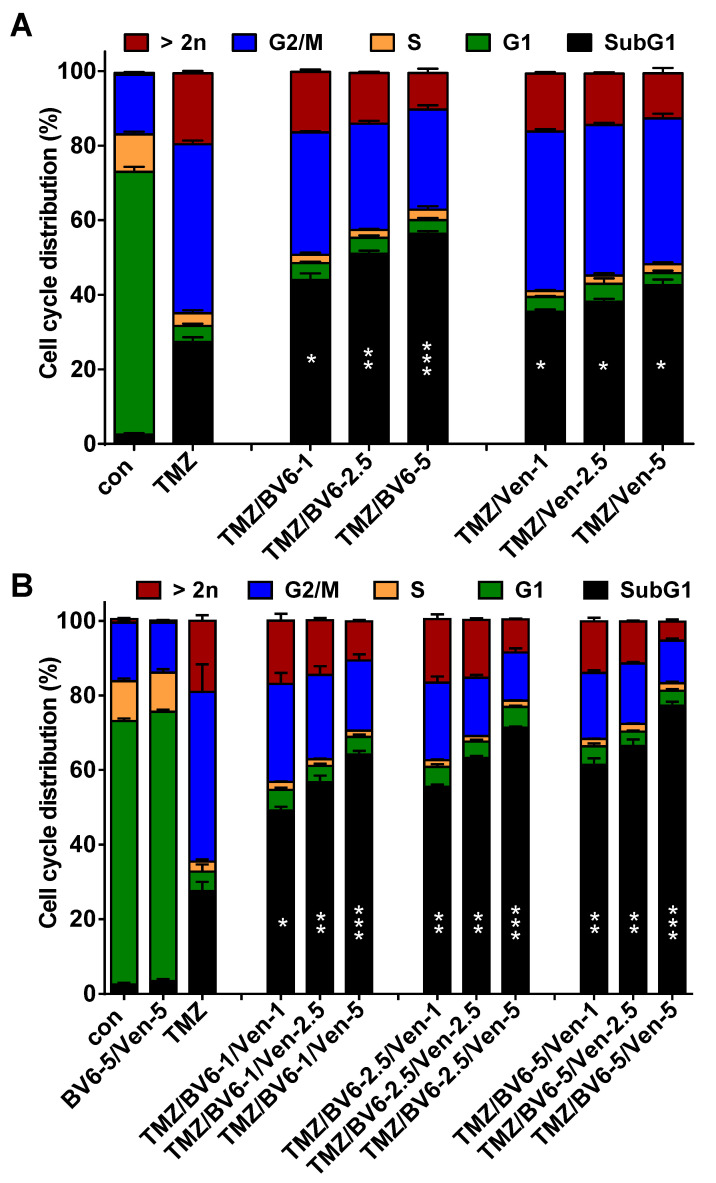
(**A**) A172 cells were treated with 50 µM TMZ for 120 h. Thereafter the medium was changed, and the cells were either further cultivated for an additional 120 h in the absence (TMZ) or the presence of different concentrations of BV6 or venetoclax (TMZ/inhibitor). As a control, cells were cultivated for 120 h; thereafter, the medium was replaced, and the cells were further cultivated for 120 h (con). After a total of 240 h, cell death and cell cycle distribution were measured by flow cytometry using PI staining. (**B**) A172 cells were treated with 50 µM TMZ for 120 h. Thereafter, the medium was changed, and the cells were either further cultivated for an additional 120 h in the absence (TMZ) or the presence of 1, 2.5, or 5 µM BV6 combined with 1, 2.5, or 5 µM venetoclax. As control, the cells were cultivated for 120 h; thereafter, the medium was replaced, and the cells were further cultivated for 120 h in the absence (con) or presence of the inhibitors (5 µM BV6/5 µM venetoclax). After a total of 240 h, cell death and cell cycle distribution were measured by flow cytometry using PI staining. A/B experiments were repeated at least three times; mean values ± SD are shown. Differences in the SubG1 fraction between inhibitor/TMZ treatment and TMZ were statistically analyzed using Student’s *t*-test (not labeled = not significant, * *p* < 0.1, ** *p* < 0.01, *** *p* < 0.001).

**Figure A6:**
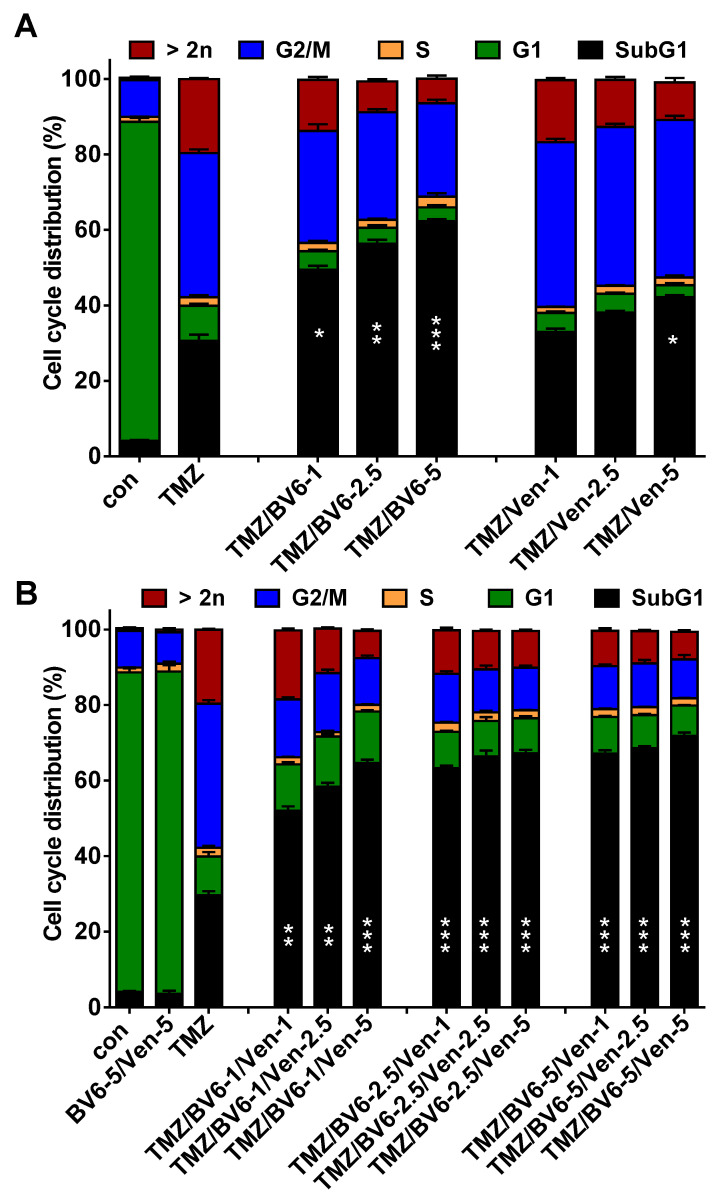
(**A**) U87MG cells were treated with 50 µM TMZ for 120 h. Thereafter, the medium was changed, and the cells were either further cultivated for an additional 120 h in the absence (TMZ) or the presence of different concentrations of BV6 or venetoclax (TMZ/inhibitor). As a control, cells were cultivated for 120 h; thereafter, the medium was replaced, and the cells were further cultivated for 120 h (con). After a total of 240 h, cell death and cell cycle distribution were measured by flow cytometry using PI staining. (**B**) U87MG cells were treated with 50 µM TMZ for 120 h. Thereafter, the medium was changed, and the cells were either further cultivated for an additional 120 h in the absence (TMZ) or the presence of 1, 2.5, or 5 µM BV6 combined with 1, 2.5, or 5 µM venetoclax. As a control, cells were cultivated for 120 h; thereafter, the medium was replaced, and the cells were further cultivated for 120 h in the absence (con) or presence of the inhibitors (5 µM BV6/5 µM venetoclax). After a total of 240 h, cell death and cell cycle distribution were measured by flow cytometry using PI staining. A/B experiments were repeated at least three times; mean values ± SD are shown. Differences in the SubG1 fraction between inhibitor/TMZ treatment and TMZ were statistically analyzed using Student’s *t*-test (not labeled = not significant, * *p* < 0.1, ** *p* < 0.01, *** *p* < 0.001).

**Figure A7:**
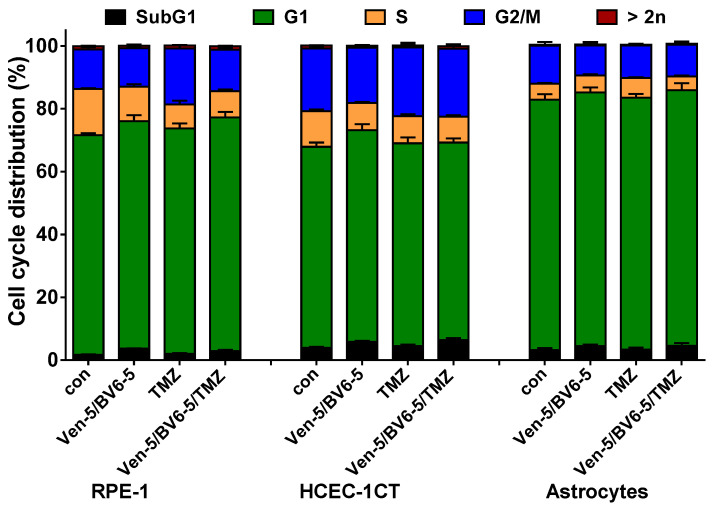
RPE-1, HCEC, and telomerase immortalized astrocytes were non-treated (con), treated with 50 µM TMZ or 5 µM BV6 + 5 µM venetoclax or 50 µM TMZ + 5 µM BV6 + 5 µM venetoclax. After a total of 120 h, cell death and cell cycle distribution were measured by flow cytometry using PI staining. Experiments were repeated three times; mean values ± SD are shown. Differences in the SubG1 were statistically analyzed using Student’s *t*-test and were found to be not significant.

To analyze whether BV6 and venetoclax also enhance the killing effect in A172 and U87MG cells, the cells were exposed to 50 µM TMZ for 120 h to induce senescence. Thereafter, the medium was replaced with fresh, FBS-containing medium, and the cells were left either untreated or were treated with the inhibitors, either single or combined. The data show that, similar to LN-229 cells, a clear dose-dependent increase of toxicity was observed for all combinations of BV6 and venetoclax. In detail, cell death frequency was enhanced in A172 cells from 27% to ~ 50% at the lowest concentrations (1 µM BV6/1 µM venetoclax) and to ~ 80% at the highest concentrations (5 µM BV6/5 µM venetoclax) (Figure A5), and in U87MG cells cytotoxicity was increased from 29% to ~ 60% at the lowest concentrations (1 µM BV6/1 µM venetoclax) and to ~ 8 0% at the highest concentrations (5 µM BV6/5 µM venetoclax) (Figure A6). In addition, for U87MG and A172 cells, the statistical evaluation using Combenefit analysis showed a significant synergy between BV6 and venetoclax in the killing of senescent cells (Figure 5E and Figure 6E). Similar to LN229, the combination of 5 µM BV6 and 5 µM venetoclax was not toxic by itself in U87MG and A172 cells. Of note, also in telomerase immortalized human epithelial cells (RPE-1 and HCEC-1CT), as well as in telomerase immortalized human astrocytes, this treatment was not toxic, either in the absence or presence of TMZ (Figure A7).

Overall, the presented data clearly supports our hypothesis that c-IAP2 and Bcl-2 act as SCAP factors in TMZ-induced senescence, and that inhibition of these factors could be used to enhance TMZ-triggered cell death of glioma cells.

## 4. Discussion

Therapy of high-grade gliomas rests on treatment with the methylating anticancer drug TMZ. However, at treatment-relevant doses, only a minor fraction of tumor cells is killed by apoptosis; malignant glioma cells rather undergo senescence [7,8]. This is likely the reason why TMZ treatment can only extend the median survival of patients from 12.1 up to 14.6 months [2]. Induction of apoptosis and senescence by TMZ is triggered *via* induction of the minor DNA lesion *O^6^*MeG and subsequent activation of the DDR [45]. Using synchronized cells, it was shown that *O^6^*MeG triggers accumulation of cells in the G2/M-phase of the post-treatment cell cycle [46]. Moreover, we could show that TMZ-induced senescence is associated with a strong NF-κB dependent induction of the SASP phenotype [8]. Since senescent cells may represent a threat for a patient during therapy caused by the SASP or by potential senescence evasion and associated increase in aggressiveness, targeting senescent cells during or after TMZ treatment could be therapeutically beneficial [35].

Since elimination of senescent cells by senolytic drugs may be cell-type specific [47], we performed our study in three different glioblastoma cell lines. Treatment of LN-229, A172, and U87MG cells with 50 µM TMZ for 120 h induced cell death only up to 20%. At the same time, up to 70, 45, or 65% of senescent cells, respectively, were induced by the drug. Of note, also for A172, the amount of senescent cells increased up to 75% at later post-exposure times. In all cell lines, senescence was accompanied by arrest in the G2-phase of the cell cycle, which confirms previous reports [8,48]. All cell lines showed the emergence of polyploid cells (20–30% of the population) upon treatment with 50 µM TMZ and a post-exposure time of 120 h. Very recently, we reported that glioma cells showing a strong accumulation of nuclear Survivin also exhibit an increased polyploid cell population after exposure to TMZ [48]. This indicates that a high frequency of cells was able to undergo incomplete cell division. Since most of the cells are tetraploid (4n), the data indicate that these cells have completed mitosis but failed to undergo cytokinesis, most likely due to chromosome mis-segregation. It has been shown that abnormal mitosis triggers p53-dependent cell cycle arrest in human tetraploid cells [49]. Therefore, we suppose that tetraploidy is also involved in triggering and maintaining TMZ-induced senescence. It has also been shown that during senescence, polyploidization is associated with re-replication of damaged G2/M cells, which could be associated with senescence-specific occurrence of heterochromatinization [50]. Of note, these tetraploid cells were also found among senescence-escaping cells. On the other hand, during carcinogenesis, senescence can block cell transformation of normal cells due to a specific G1-checkpoint in tetraploid cells [51].

The idea of using agents that specifically kill senescent cells, i.e., senolytic drugs [52], is based on the observation that senescent human fibroblasts resist apoptosis [53]. In this early study, the involvement of anti-apoptotic pathways was established, suggesting that Bcl-2 is important for apoptosis evasion of senescent cells. This led to the positing of a “Senescent Cell Anti-Apoptotic Pathway (SCAP)”, which is important for blocking apoptosis and thereby triggering senescence [54]. Based on the dominant role of anti-apoptotic factors in the SCAP, we analyzed which factors are involved in apoptosis evasion in TMZ-induced senescence of glioblastoma cells. Thus, we analyzed expression of the anti-apoptotic factors Bcl-2, Bcl-xL, c-IAP1, c-IAP2, XIAP, and Survivin and elucidated whether inhibition of these factors could enhance cell killing by targeting senescent cells.

**Figure A8:**
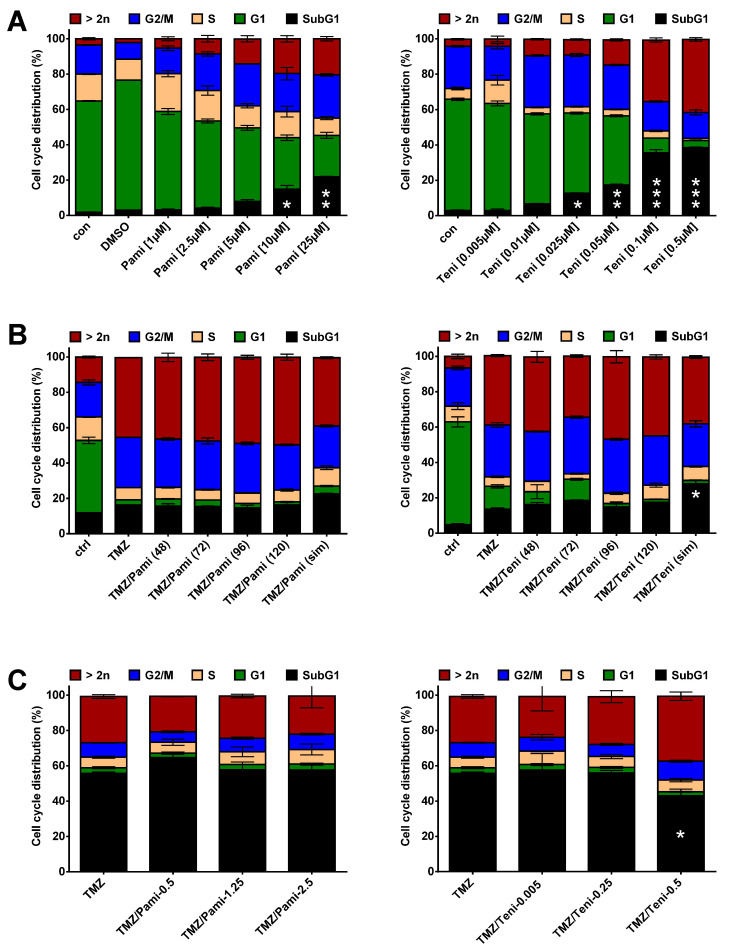
(**A**) LN-229 cells were treated with different concentrations of the topoisomerase II inhibitor teniposide (Teni) or the PARP inhibitor pamiparib (Pami). Cell death and cell cycle distribution was measured by flow cytometry using PI staining. Experiments were repeated at least three times; mean values ± SD are shown. (**B**) LN-229 cells were either non-treated (con) or treated with 50 µM TMZ for 120 h (TMZ). In addition, 0.025 µM teniposide or 2.5 µM pamiparib were added either simultaneously with TMZ (sim) or 48, 72, or 120 h after TMZ exposure. Cell death and cell cycle distribution was measured by flow cytometry using PI staining in LN-229 cells. Experiments were repeated at least three times; mean values ± SD are shown. (**C**) LN-229 cells were treated with 50 µM TMZ for 120 h. Thereafter the medium was changed, and cells were again treated with 50 µM TMZ for 120 h (TMZ) or with TMZ combined with different concentrations of teniposide or pamiparib. (**A**–**C**) Experiments were performed in triplicate, and differences of cells in SubG1 between Pami or Teni vs. control (**A**), as well as TMZ/Pami or TMZ/Teni vs. TMZ alone (**A**,**B**) were statistically analyzed using Student’s *t*-test (not labeled = not significant, * *p* < 0.1, ** *p* < 0.01, *** *p* < 0.001).

Here we show that TMZ induced a strong transcriptional activation of *c-IAP2* and a weak induction of *c-IAP1* and *XIAP* in LN229, A172, and U87MG cells. In U87MG cells, *Bcl-2* and *Bcl-xL* were also transcriptionally activated. The induction of c-IAP2 was also observed at the protein level. Furthermore, Bcl-2 also showed increased protein expression, which seems to be regulated by post-transcriptional mechanisms. Overall, the data show a strong activation of anti-apoptotic factors. Therefore, we analyzed whether inhibition of these factors enhances TMZ-induced cell death at early time points (senescence induction) and at later times (senescence maintenance). The data reveal that BV6, predominantly targeting c-IAP1/c-IAP2 and to a lesser extent XIAP (but not venetoclax, targeting Bcl-2/ embelin, targeting XIAP / or AT406, targeting c-IAP1), significantly increased toxicity already at early time points. Similar data were also observed during late stages of senescence. In these experiments, cells were exposed to 50 µM TMZ for 120 h to induce senescence and after medium replacement exposed to TMZ or to the inhibitors for an additional 120 h. Interestingly, at this time point, e.g., 240 h after TMZ exposure, the cell death of the TMZ-only treated cells amounted to 25–40%, indicating ongoing induction of cell death also at very late times after TMZ exposure. We should note that additional treatment with TMZ was not able to enhance toxicity. The same was also observed upon exposure to the topoisomerase II inhibitor teniposide and the PARP inhibitor pamiparib (Figure A8). In contrast, treatment with non-toxic concentrations of BV6 and venetoclax, but not with AT406 and embelin, eradicated the senescent cells.

Our data clearly show that SCAP inhibition efficiently kills TMZ-treated cells. However, it is not possible to formally prove that only senescent cells were killed, i.e., that the killing was a purely senolytic effect. Our data reveal that the inhibition of c-IAP1/2 and of Bcl-2 also kills cells at early time points after TMZ exposure. After all, this is to be expected, since the SCAP is a prerequisite for the onset of senescence. Thus, inhibition of the SCAP can also trigger cell death at early time points before development of the senescent phenotype in TMZ-exposed cells. In addition, 120 h after TMZ exposure, cells not yet displaying a senescent phenotype could therefore be killed. It is however more likely that particularly cells already showing the senescent phenotype are killed. This is supported by the fact that in colony-forming assays, only 0.02% of LN-229 cells were able to proliferate and is also substantiated by the finding that 240 h after TMZ exposure, nearly 100% of the surviving cells are senescent (data not shown).

**Figure A9:**
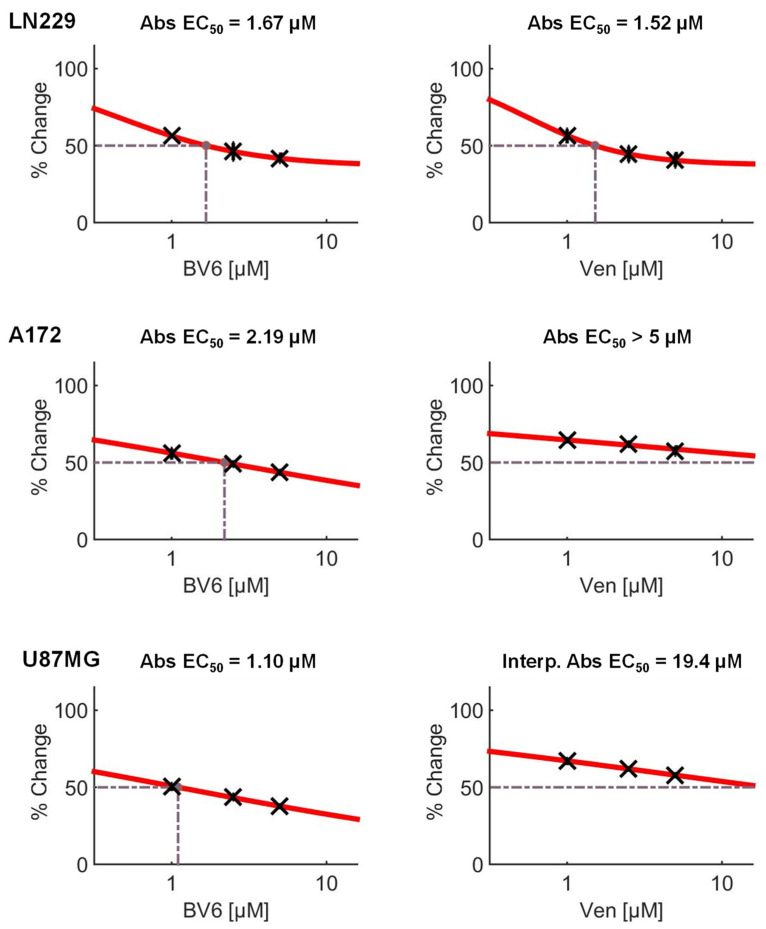
Toxicity data presented in Figure 4A, Figure A5A and Figure A6A were used to calculate EC_50_ of BV6 and venetoclax using Combenefit software.

The killing effect of BV6 and venetoclax could even be enhanced by combining these drugs, as indicated by the observed synergistic effect and the statistically evaluated Loewe-synergy, calculated via Combenefit. Comparing the efficiency of BV6 and venetoclax in all three cell lines revealed that BV6 is more effective in killing senescent cells. This is also reflected by the low EC_50_ for BV6 (LN-229: 1.67 µM; A172: 2.19 µM; U87MG: 1.1 µM) (Figure A9). For venetoclax, a low EC_50_ was only observed in LN-229, but not in A172 (>5 µM) and U87MG (19.4 µM) cells.

The importance of Bcl-2 as a mediator of SCAP was also reported during doxorubicin-induced senescence in breast cancer cells. Here, the Bcl-2/Bcl-xL/Bcl-w inhibitor ABT-263 efficiently killed senescent cells [55]. Moreover, senescent prostate cancer cells, accumulating DNA damage induced by ionizing radiation and PARP inhibition, were killed by the Bcl-2 inhibitors navitoclax and ABT-737 [56]. In contrast, upon TMZ-induced senescence, c-IAP2 seems to play a prevailing role in mediating the SCAP, which was also indicated by the Combenefit analyses. Of note, our data are in line with those showing that BV6 can sensitize glioblastoma cells to TMZ [57] and that *BIRC3/c-IAP2* up-regulation results in apoptosis evasion and therapeutic resistance in glioblastoma [58]. Moreover, concerning the impact of IAP inhibition on anticancer drug therapy, we could already show that colorectal cancer (CRC) cells could be sensitized to irinotecan by the Survivin inhibitor LLP3 [59,60] and, most importantly, BV6 also sensitized CRC cells to irinotecan [61].

We should note that an unresolved problem concerning in vitro studies using TMZ is based on the applied concentrations [62]. Whereas the median serum concentration of TMZ achieved is 50 µM [63], the data are less clear for the cerebrospinal fluid. Thus, peak concentrations in the cerebrospinal fluid are in the range of 14.95–34.54 µM [64], 0.82–9.94 µM [65], 1.55–4.64 µM [66], and 1.49–4.17 µM [67]. In vitro, among 12 glioma cell lines, only LN-229 had an EC_50_ < 100 µm in the acute cytotoxicity assay [68]. Opposite, concerning clonogenic survival, most cell lines have an EC_50_ < 100 µm. This suggests that under clinical conditions, TMZ is not cytotoxic but rather cytostatic [69]. Additionally, in vitro, TMZ predominantly induced senescence and not cell death [8]. In our experiments, only at late time points a significant TMZ-induced toxicity was observed in A172 and U87MG cells. LN-229 showed a higher frequency of cell death at 25 and 50 µM, whereas concentrations of 5 and 10 µM were not toxic but strongly affected clonogenic survival in all cell lines (present publication, [8]). It is important to note that in the low concentration ranges between 1 and 25 µM TMZ, a linear increase with dose in the activation of the DNA damage response, cell death, and senescence was observed without a no-effect threshold [43,44]. For in vitro experiments, higher concentrations are often necessary, since established cell lines are more robust and not as responsive as cells in vivo. This is already the case for freshly established glioblastoma tumor spheres [70]. Nevertheless, in these tumor spheres, responders and non-responders could be separated in vitro, and a direct relationship was found between sensitivity of tumor spheres to TMZ and patients’ survival [70]. The increased resistance to TMZ in vitro can be ascribed to multiple adaptive events in cell culture, e.g., differentiation that gradually shifts terminally differentiated cells to a post-mitotic state [71], as well as to epigenetic alterations or even to pro-survival serum factors. In summary, the fact that the concentrations used for in vitro experiments are higher than those observed in vivo should not prevent pre-clinical work to uncover pathways that could be targeted to improve the therapeutic response of patients. In addition, other application schedules, such as Ommaya reservoirs could achieve higher concentrations of the drugs used [72]. Therefore, it would be highly important to include inhibitors of c-IAP2 and Bcl-2 in future studies using orthotopic models and to develop more efficient and blood–brain barrier-penetrating drugs.

## 5. Conclusions

Overall, our data presented here and previously published indicate that in glioblastoma cells, TMZ induces senescence to a significantly higher level than cell death through apoptosis and necrosis [7,8,43,48]. Senescence is associated with activation of several anti-apoptotic factors, such as Bcl-2, c-IAP1, and c-IAP2. The Bcl-2-inhibitor venetoclax and the c-IAP1/c-IAP2 inhibitor BV6 can enhance TMZ toxicity at early times after TMZ treatment and, most importantly, also target senescent glioblastoma cells at later times. Our data indicate that Bcl-2 and c-IAP2 represent the core of the SCAP in TMZ-induced senescence and, therefore, point to the need for in vivo studies, targeting these factors to improve TMZ-based glioblastoma therapy.

## Data Availability

Data is contained within the article or Supplementary Material.

## Supplementary Materials

The following are available online at https://www.mdpi.com/article/10.3390/cancers13143585/s1, Figure S1: Uncut immunodetection generated by direct scanning the area of interest using the iBright CL1000 (Invitrogen) system.

## Appendix A

**Table A1:** Primer sequences used for qPCR.

Gene	Primer Sequence (5′-3′)
*ACTB-fw*	TGGCATCCACGAAACTACC
*ACTB-rev*	GTGTTGGCGTACAGGTCTT
*Bcl2-fw*	TTCAGAGACAGCCAGGAGAAA
*Bcl2-rev*	AGTACCTGAACCGGCACCT
*BCLxL-fw*	AAGCGTAGACAAGGAGAT
*BCLxL-rev*	TAGGTGGTCATTCAGGTAA
*BIRC2/c-IAP1-fw*	TTCCCAGGTCCCTCGTATCA
*BIRC2/c-IAP1-rev*	CCGGCGGGGAAAGTTGAATA
*BIRC3/c-IAP2-fw*	TCACTCCCAGACTCTTTCCA
*BIRC3/c-IAP2-rev*	CCCCGTGTTCTACAAGTGTC
*BIRC5/survivin-fw*	ATGACTTGTGTGTGATGA
*BIRC5/survivin-rev*	GTTTGTGCTATTCTGTGAA
*CDNK1a-fw*	TACATCTTCTGCCTTAGT
*CDNK1a-rev*	TCTTAGGAACCTCTCATT
*GAPDH-fw*	CATGAGAAGTATGACAACAG
*GAPDH-rev*	ATGAGTCCTTCCACGATA
*XIAP-fw*	CCGAAGAGAAACCACATTT
*XIAP-rev*	CTGAGCCAGATCAAAGTATG

**Table A2:** Antibodies used for immunodetection.

Protein	Molecularweight (kDa)	Antibody	RRID	Company
ß-Actin	42	sc-47778	AB_2714189	Santa Cruz Biotechnology
c-IAP1	70	7065S	AB_10890862	Cell Signaling Technology
c-IAP2	68	3130S	AB_10693298	Cell Signaling Technology
BCL-2	26	4223	AB_1903909	Cell Signaling Technology
BCL-xL	26	2764	AB_2228008	Cell Signaling Technology
Survivin	16	2808S	AB_2063948	Cell Signaling Technology
XIAP	57	14334S	AB_2784533	Cell Signaling Technology
HRP conjugated anti-mouse		KCB002	AB_10703407	Rockland
HRP conjugated anti-rabbit		KCB003	AB_10702763	Rockland
