# Supplementary material for: Targeting c-IAP1, c-IAP2, and Bcl-2 Eliminates Senescent Glioblastoma Cells Following Temozolomide Treatment
Source: Cancers (Basel). 2021 Jul 17;13(14):3585. doi: 10.3390/cancers13143585 (PMC8306656; doi:10.3390/cancers13143585)
Supplement: Supplementary file 1 [file cancers-13-03585-s001.zip › cancers-1281037-supplementary.pdf]

If not stated otherwise immunodetection was performed using the iBright CL1000 (Invitrogen) system, directly scanning the area of interest.

Presented scans were not processed by additional software.

Dotted lines show the size of the scanned area.

Detection of the protein of interest and loading controls (HSP90/b-Actin) were performed on the same membrane.

In some cases, the membrane was cut, to perform detection of two different proteins at the same time.

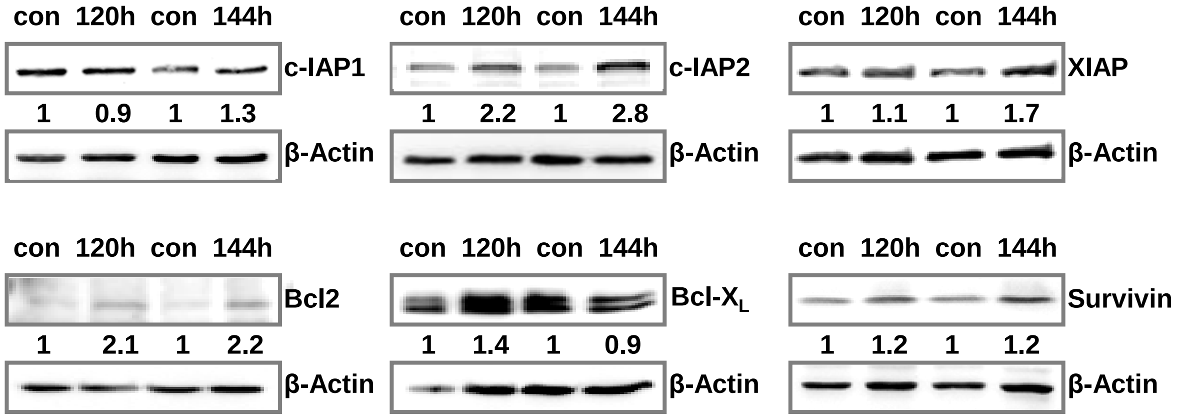

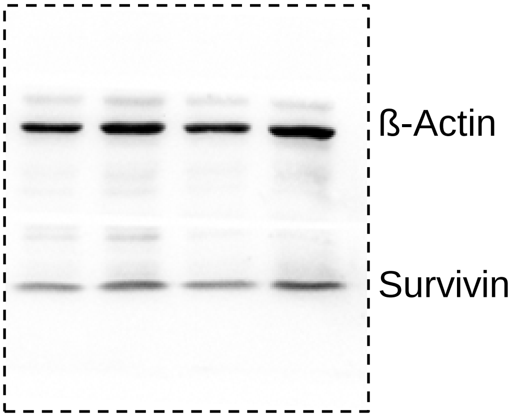

Membran cut
Immunodetection
performend in parallel

---

* Lane empty (air bubble in gel)

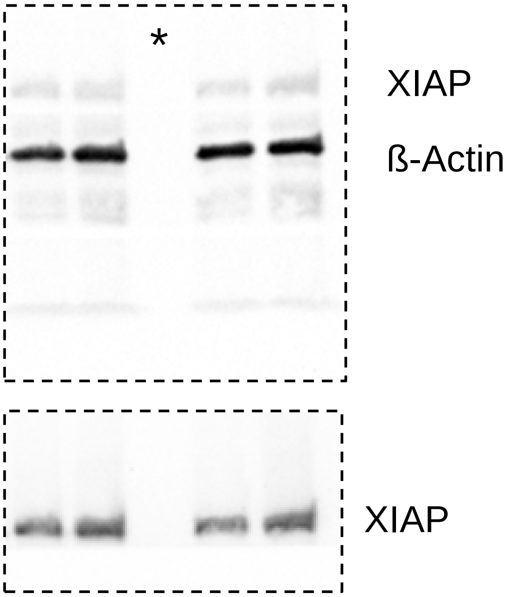

Membran cut
Immunodetection
performend in parallel

Higher exposure
membrane part with XIAP

c-IAP1

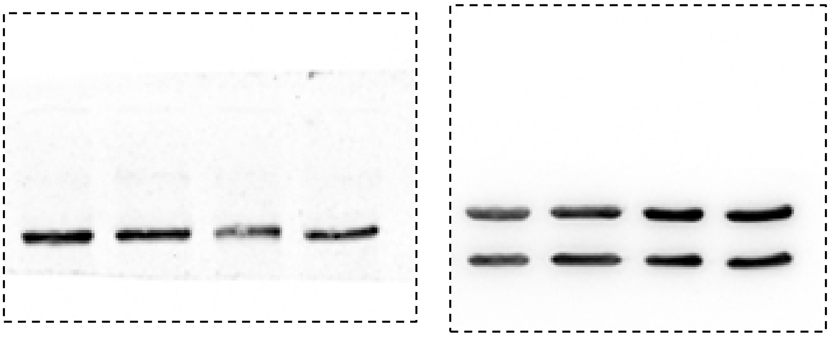

ß-Actin
GAPDH

Membran cut and immunodetection performend in parallel

c-IAP2

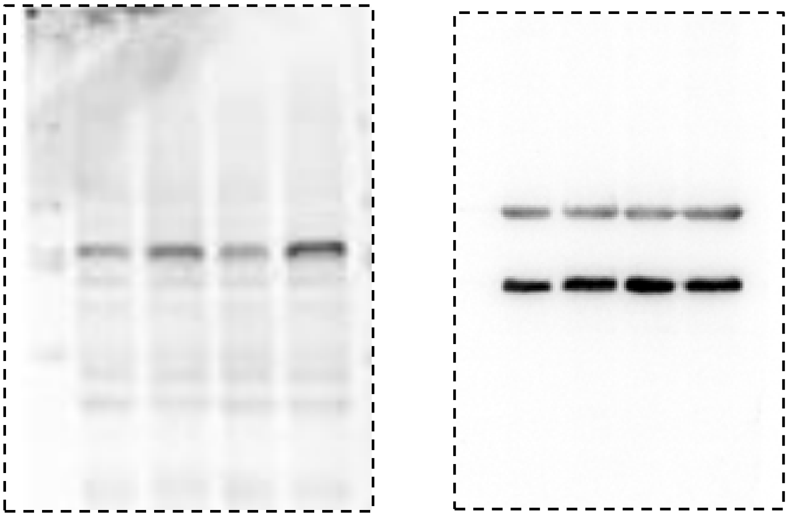

HSP90
ß-Actin

Bcl2

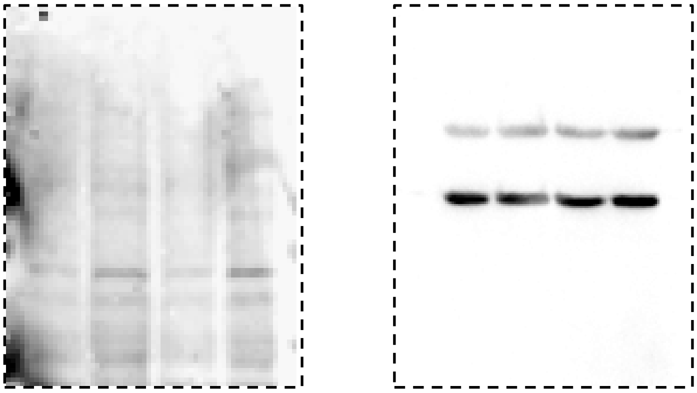

HSP90
ß-Actin

Bcl_{XL}

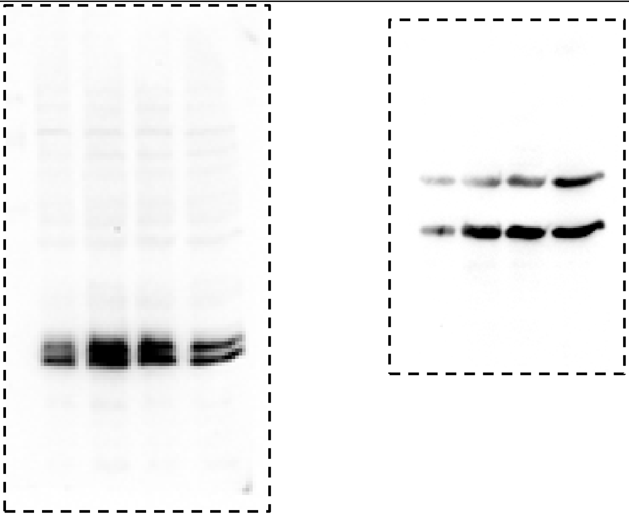

HSP90
ß-Actin
